# Protecting vulnerable communities and health professionals from COVID-19 associated mental health distress: a comprehensive approach led by a public-civil partnership in rural Chiapas, Mexico

**DOI:** 10.1080/16549716.2021.1997410

**Published:** 2021-12-10

**Authors:** Ana Cecilia Ortega, Erika Valtierra, Fátima Gabriela Rodríguez-Cuevas, Zeus Aranda, Gisela Preciado, Sebastián Mohar

**Affiliations:** a Mental Health Program, Partners In Health Mexico/Compañeros En Salud, Ángel Albino Corzo, México; b Department of Global Health and Development, London School of Hygiene and Tropical Medicine, London, UK; c Research and Impact Program, Partners in Health Mexico/Compañeros En Salud, Ángel Albino Corzo, México; d Hospital Básico Comunitario de Ángel Albino Corzo, Chiapas Ministry of Health, Ángel Albino Corzo, México

**Keywords:** Psychosocial support, capacity building, low- and middle-income countries, task-sharing, community mental health

## Abstract

**Background:**

The COVID-19 pandemic has stricken mental health worldwide. Marginalized populations in low- and middle-income countries have been the most affected, as they were already experiencing barriers to accessing mental health care prior to the pandemic and are unequally exposed to the stressors associated with the health emergency, such as economic ravages or increased risk of complicated disease outcomes.

**Objective:**

The aim of this paper is to describe a comprehensive initiative resulting from a public-civil partnership to address the increased burden of mental health illness associated with the COVID-19 pandemic in rural Chiapas, Mexico.

**Methods:**

To address the emerging health needs of the general population and health professionals resulting from the pandemic, Compañeros En Salud (CES), a non-profit civil society organization based in Chiapas, implemented a comprehensive strategy to compensate for the shortage of mental health services in the region in collaboration with the Chiapas Ministry of Health. The strategy included three components: capacity building in mental health care delivery, psychosocial support to the general population, and provision of mental health care to CES collaborating staff. In this capacity building article, implementers from CES and the government share descriptive information on the specific interventions carried out and their beneficiaries, as well as a critical discussion of the strategy followed.

**Results:**

Through this strategy, we have been successful in filling the gaps in the public health system to ensure that CES-served populations and CES-collaborating health professionals have access to mental health care. However, further studies to quantify the impact of this intervention in alleviating the burden of mental health illnesses associated with the pandemic are needed.

**Conclusions:**

The current situation represents an opportunity to reimagine global mental health. Only through the promotion of community-based initiatives and the development of integrated approaches will we ensure the well-being of marginalized populations.

## Background

COVID-19 has affected health, social, and economic matters in devastating ways, negatively impacting mental health worldwide [1]. Research shows that the pandemic has increased levels of depression, anxiety, insomnia [2,3], post-traumatic stress disorder [4], and suicide ideation amongst the general population [5], as well as psychological distress amongst frontline health workers [6,7]. Increased demand for mental health care has coincided with the disruption of mental health services; according to a survey conducted by the World Health Organization (WHO) in mid-2020, mental health services had been disrupted by the pandemic in 93% of the 130 participating countries [8].

Evidence shows that financially vulnerable populations in low-and-middle income countries (LMICs) have a higher risk of developing mental health conditions related to the pandemic. In LMICs, individuals have more impediments to practicing social distancing, face barriers to accessing health care, and are more likely to be affected by the economic impacts of lockdowns [9].

As COVID-19 threatens to widen the mental health treatment gap in LMICs, rural communities with profound barriers to access healthcare are most likely to suffer. Chiapas, the southernmost state in Mexico and one of its poorest, with half of its population living in rural areas [10], has high rates of mental health disorders and limited health services. Before the pandemic, the prevalence of depression in the Fraylesca and Sierra regions – the areas that we serve – was estimated at 7.9% [11], whereas the Mexican national average remained in 4.0–4.5% [12]. As for younger populations, 35.8% of adolescents suffer from depression or generalized anxiety disorder, and 32.1% with both mental disorders report having attempted suicide [13]. Furthermore, the lifetime prevalence of Intimate Partner Violence (IPV) in the region is 54.7% among ever-partnered women, with significant associations between depressive symptoms and both IPV and Sexual Violence (SV) [14].

Several interventions are being undertaken to address mental health in LMICs during the pandemic [9], but attention is still focused on the Global North [15]. In this piece, we depict a series of mental health interventions implemented from March 2020 to February 2021 by the Ministry of Health and the non-profit organization Compañeros en Salud (CES) in rural Chiapas. This is not an empirical research paper, but a description and discussion of the integration of mental health services during the COVID-19 pandemic, aiming to expand the literature on mental health interventions in low resource settings.

### The public health sector response to the mental health burden associated to the pandemic

With the advent of the COVID-19 pandemic, many of the health services were reimagined and adapted to meet the population’s needs and to respond to the suspension of services and reduction of personnel in the Mexican healthcare system. Chiapas’ Ministry of Health (MoH) transformed 14 clinical spaces – ranging from vaccination centers to abandoned Intensive Care Units – into COVID-19 clinics [16]. However, providing mental health care in the midst of a pandemic with an already deficient system was challenging. In the Mexican public health sector, only 2% of healthcare expenditure is allocated to mental healthcare with 80% going to hospitals, leaving the primary care level underserved [17].

In June 2020, a Post-COVID Psychology Rehabilitation Center was inaugurated in Tuxtla Gutiérrez, the capital city of Chiapas, to provide mental healthcare for patients, their relatives, and front-line health providers affected by COVID-19. In addition, 93 psychosocial hotlines were created for crisis management and emotional support [18]. However, these services were not available for those living in rural areas or those lacking mobile phone reception, such as the Sierra and Fraylesca regions. Although the local health district attempted to provide coping tools and burnout prevention skills to the staff in the clinics, there was no concrete plan for the provision of mental health support at COVID-19 clinics.

### Compañeros En Salud: a key player in addressing mental health disparities exacerbated by the pandemic

**Figure 1.**
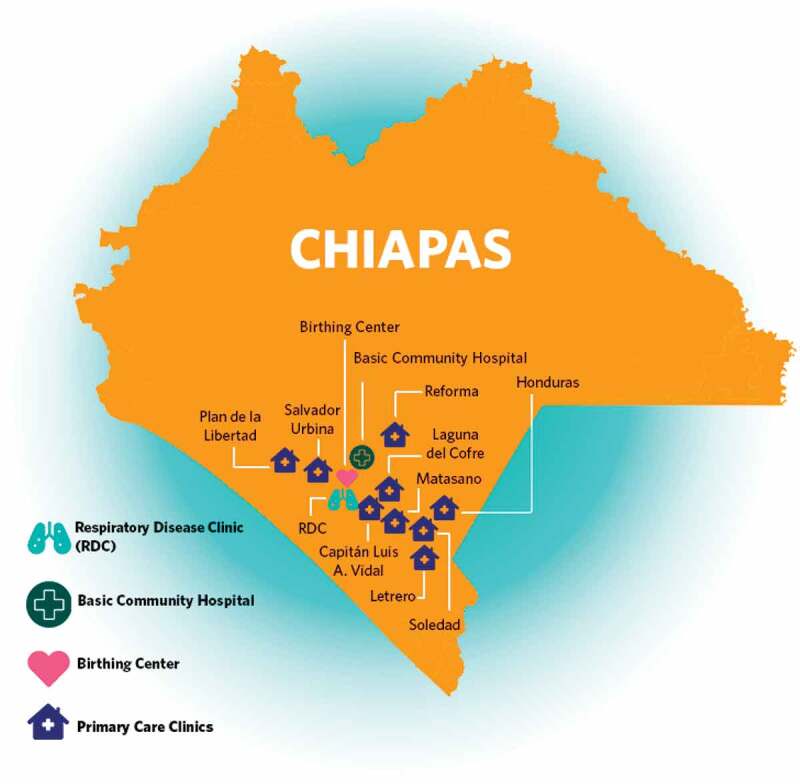
Facilities supported by Compañeros En Salud in Chiapas, México

CES is the Mexican sister organization of the international non-profit organization Partners In Health (PIH), and has been working in collaboration with the MoH since 2011 to strengthen the healthcare system in Mexico. CES provides access to high quality services by working in ten MoH primary care clinics in nine rural communities and in a birthing center, a basic community hospital and, most recently, a Respiratory Disease Clinic (RDC) in the small town of Ángel Albino Corzo, all located in the Fraylesca and Sierra regions of Chiapas (see Figure 1).

In 2014, CES launched the mental health program in response to the high burden of mental health conditions in the area. The program is based on task-sharing [19], stepped care [20], and collaborative care models [21]. The activities of the mental health program are embedded in other CES projects, which consist of sexual and reproductive health, community health, referral to specialized services, continuous medical education, community-based mental healthcare (psychosocial and pharmacological treatments), and staff wellbeing.

In CES’ catchment area, there were several factors that helped to identify that these communities were at a potential risk of developing mental health conditions related to COVID-19: 1) financial instability, 2) lack of access to mental health services, and 3) previous high prevalence of mental health conditions. These factors justified the need for a preventative and therapeutic psychosocial intervention.

**Figure 2.**
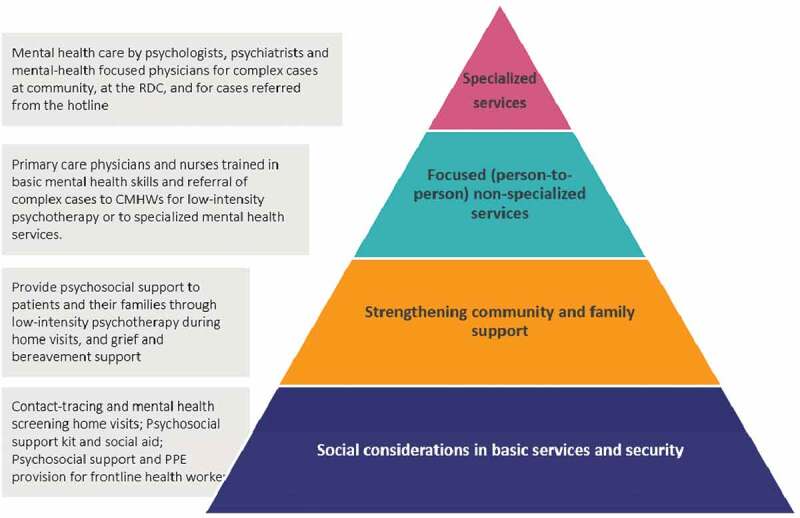
Psychosocial support services. Adapted from the Inter-Agency Standing Committee’s Intervention Pyramid for mental health and psychosocial support in emergencies [23]. RDC: Respiratory Disease Clinic, CMHWs: community mental health workers, PPE: personal protection equipment

In response, CES launched a mental health intervention aligned with PIH’s broader initiative called ‘STOPCOVID,’ which aimed to prepare vulnerable populations to combat COVID-19 in contexts where social distancing and safe quarantine and isolation are oftentimes not feasible [22]. We will present three strategies that comprise CES’ mental health response to COVID-19 based on the Inter-Agency Standing Committee’s Guidelines on Mental Health and Psychosocial Support in Emergency Settings (see Figure 2) and on the following principles: a) capacity building, b) psychosocial support, and c) staff mental health care [23].

## Capacity building

A fundamental aspect of CES’ COVID-19 response was the training of non-specialists in mental health skills for meeting the emotional needs at the community and in-patient unit levels.

### Psychological First Aid training for all staff members

All the clinical and non-clinical staff were trained on Psychological First Aid (PFA), an intervention that provides immediate support to individuals experiencing distress related to a recent crisis. PFA can be provided by anyone who has received proper training and who can make referrals to mental health professionals when required [24]. Although systematic reviews report that there is no direct evidence of PFA effectiveness, indirect evidence supports its delivery in a variety of settings [25,26].

**Figure 3.**
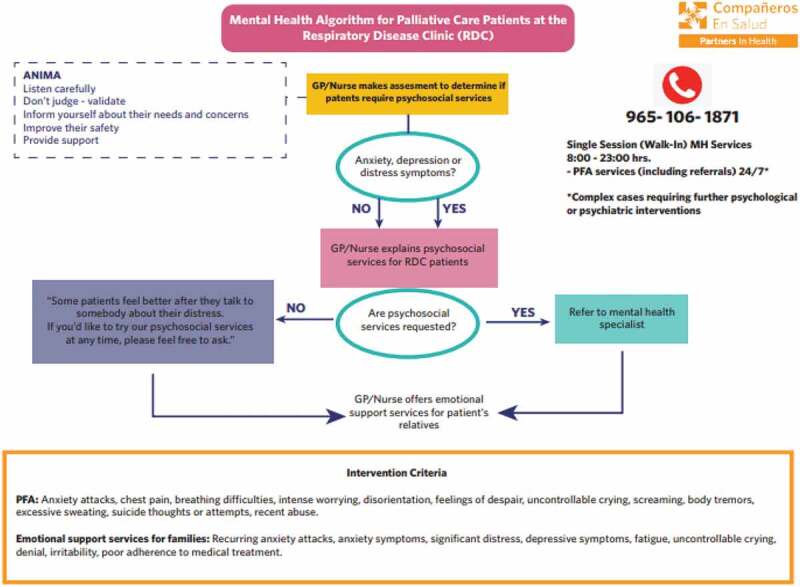
Pathway of care of COVID-19 response at the Respiratory Disease Clinic. GP: general practitioner, MH: mental health, PFA: Psychological First Aid

Training was provided to 292 people, both from CES and the MoH, from March 2020 to January 2021. These PFA workshops were based on the WHO’s PFA orientation guidelines [27] and included a theoretical explanation of common reactions to stressful events, a practical review of relevant nonverbal cues, how to provide PFA (through modeling and peer practice), the referral system (see Figure 3), and self-care strategies. Because of the briefness of these trainings, a pocket field guide was designed for community staff, which was culturally adapted for both community health workers (CHWs) and community mental health workers (CMHWs).

### Psychosocial training for Respiratory Disease Clinic staff

Besides the PFA training, physicians and nurses from the RDC were trained in the following topics: basic psychosocial support [28], effective communication for conflict prevention and management, mental health referral criteria, bereavement and thanatological support, occupational therapy and COVID-19 rehabilitation [29].

### Training for Community Health Workers and adolescent volunteers

Currently, there are more than 80 CHWs and five CMHWs working in CES-supported rural communities. As a response to the increasing cases of COVID-19 in the communities, 44 CHWs and all of the CMHWs were trained in contact tracing. The home-visit questionnaire included three questions that explored mood, anxiety and suicidal thoughts. If people answered positively to the latter, they would be referred to a CMHW for further evaluation. Though both the CHWs and CMHWs were trained in grief and bereavement, CMHWs received a more in-depth training on this topic [30]. In addition, 11 adolescent mental health volunteers participated in an introductory training to mental health and COVID-19 with a gender perspective, allowing them to provide information to their peers and refer other adolescents with mental health conditions to the health clinic.

## Psychosocial support for the general population

This support constituted one of the pillars of CES’ mental health intervention during the pandemic, since specialized care was provided to patients with psychological needs in Ángel Albino Corzo and in the nine rural communities supported by CES.

### Respiratory disease clinic

#### Hospitalized patients

Daily visits were made to hospitalized patients to assess their mental health, provide counseling (including thanatological accompaniment) and create rehabilitation plans. A total of 94 patients benefited from this support. Additionally, during these months, 23 occupational therapy kits were provided to patients, as a way of facilitating behavioral activation and improving the psychological well-being of hospitalized patients [29]. These kits included activities such as: embroidery, reading, drawing, writing, listening to music, board games, relaxation exercises, and writing postcards for their loved ones. Refer to Figure 3 for the pathway of mental healthcare at the RDC.

When the pace of work allowed it, some of the activities were shared with the RDC health personnel, thus strengthening the doctor/nurse-patient trust relationship and reducing staff stress.

#### Communication with family members and psychoeducation

As a way of building communication bridges between the health personnel and the patients’ families at the RDC, a psychologist approached family members by providing psychoeducation and information for them to make informed decisions on issues such as hospital transfers, discharges, how to receive thanatological support, orientation on post-COVID-19 care, and strategies for adherence to treatment. In some cases, brief mental health counseling was also provided to family members.

### Community

#### Psychosocial toolkits

With the aim of fostering well-being strategies for youth and families while socially distancing, two kinds of psychosocial toolkits were created: one for adolescents and one for families. More than 100 kits were sent to the rural primary care clinics for distribution by the physician. While both toolkits included mindfulness exercises and psychoeducation information, the youth toolkit consisted of occupational activities and the family toolkit of activities to foster family cohesion during lockdown [31].

#### Material Support

Acknowledging that quarantine caused loss of working days, inability to access food supplies was a key determinant to be tackled for food security and decreasing stress. Right after people with suspected COVID-19 infection or their contacts received the recommendation to quarantine, they were screened for social support. Some of them met criteria for social vulnerability and 77 households received food packages and 179 hygiene kits as social aid. The hygiene kits included bleach and detergent, key to protecting other household members from infection.

#### Community mental health workers

CMHWs have been a crucial part of the COVID-19 mental health response as they have been providing. Problem Management Plus (PM+), a brief, low-intensity psychotherapy intervention developed by the WHO [32]. Apart from this psychological intervention, they provided mental healthcare for patients who were grieving, in addition to palliative mental healthcare, as new ways of accompanying bereaved families had to be created due to COVID-19 [33,34]. During 2020, 43 patients were attended to by CMHWs due to a COVID-19-associated mental health condition.

#### Outpatient psychological accompaniment

**Figure 4.**
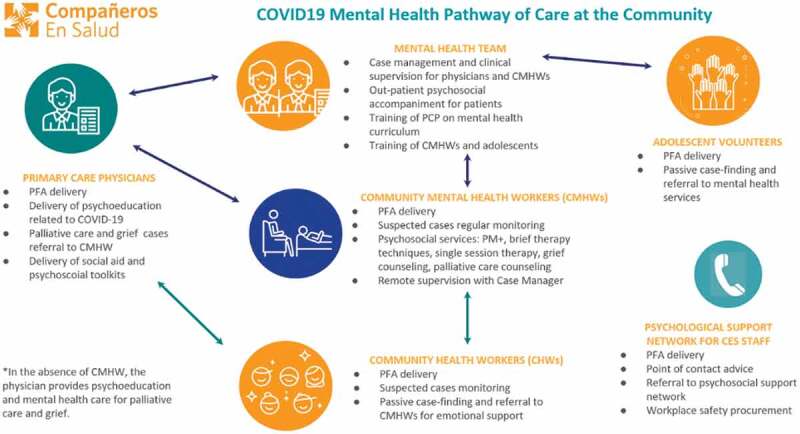
Mental health pathway of care at the community. PFA: Psychological First Aid, CMHW: community mental health workers, PCP: primary care physician

Several people began seeking psychological support, sometimes due to COVID-19-related emotional distress. In response, CES set up outpatient emotional support consultations provided by a physician and three psychologists. From April 2020 to February 2021, 89 patients have been enrolled and 203 consultations were provided. Refer to Figure 4 for the pathway of mental healthcare at the community level. Outpatient psychological accompaniment was also offered to patients residing in the town of Ángel Albino Corzo.

## Staff mental health care

Part of the strategy to fight COVID-19 included the creation of psychosocial support structures for the organization’s clinical and non-clinical staff, given that isolation and increased distress and social adversity were being experienced.

### Point of contact

Every person from the staff was allocated to a professional from the mental health team as a ‘point of contact’. The points of contact, if approached by a staff member, were instructed to offer advice around the organization’s psychosocial resources, including: a) psychological orientation, b) single session therapy, c) referral to CES´ psychosocial support network or to PIH´s remote psychosocial services, such as Konterra [35], which included virtual mental health care in Spanish. From March 2020 to February 2021, over a fifth of CES´ staff (n = 46) have approached their point of contact at least once.

### Mental health specialist network

A network of four psychologists and six psychiatrists comprised a virtual platform to which staff cases could be referred to for teleconsultations. In some cases, these experts were volunteers, and in other cases CES covered several consultations without cost for the user. These consultations included a risk assessment scale (from 0–3) that indicated whether or not the person’s own safety, the safety of others or their work duties were at risk, and if organizational support was needed. In cases where pharmacological treatment was needed, CES supplied it. During 2020, 53 people approached the network and over 155 consultations were provided.

### Virtual wellbeing groups

During 2020, six open virtual meetings for all staff members were offered, during which mental health coping techniques were provided. These meetings were facilitated by a mental health team member or by a staff member that volunteered to share a topic with the group.

### Debriefing and defusing groups

At the RDC, ongoing debriefing and defusing groups were organized with the staff members to encourage reflection on actions taken while managing complex patient care situations. Debriefing groups aimed at improving future performance and strengthening group cohesion [36], while defusing groups, opened a safe space for professionals to have emotional catharsis and relieve stress [37].

### Workplace safety

Other supportive measures were implemented, such as providing staff with personal protective equipment (PPE), training them on how to use it, protocoling COVID-19 measures, and ensuring the creation of supervision and support channels, all of which contributed to building a safe workplace. Although these measures were not directly targeted at mental health, there is evidence that they prevent emotional distress among frontline workers [38].

## Discussion

The public health sector has been overwhelmed by the sudden and exponentially increasing burden of disease in Chiapas, resulting in a difficulty to prioritize mental health in an already under-resourced system. For this reason, the MoH has collaborated with civil society organizations, such as CES, to strengthen mental health services and address the recent mental health needs associated with the COVID-19 pandemic. This collaboration is possible through an interdisciplinary team of mental health specialists, primary care health providers, and CHWs (especially CMHWs).

One of the strengths of the CES model is that it provides on-site mental health care rather than waiting for financially vulnerable patients living in rural communities to seek services. This is a necessary shift in the healthcare paradigm that we achieved by integrating mental health care into the RDC and by providing community mental health care, in the primary care clinics and by interventions delivered by CMHWs.

The high burden of mental health issues among suspected and confirmed COVID-19 patients and their family members, especially hospitalized patients, makes it crucial to provide mental health services in facilities treating COVID-19 patients [39]. Although there is little evidence for the efficacy of interventions addressing COVID-19 inpatient mental health [40], two randomized controlled trials (RCTs) conducted in China demonstrated significant improvement in anxiety symptoms using relaxation routines among these patients [41,42]. Supporting public health facilities to address the mental health needs of COVID-19 inpatients and their companions has been one the pillars of the response of CES to the pandemic, but also of other NGOs in Latin America, such as Doctors Without Borders [43].

In our work, we observed that not only did COVID-19 patients and their families experience the stress of uncertainty and the need to travel long distances to seek medical care, but also the financial burden of traveling and lodging. Therefore, intersectoral supportive programs that address the out-of-pocket expenses of patients and their families need to be contemplated. Once patients and their families return home, maintaining social support is critical to encourage people to maintain quarantine and prevent food insecurity related stress.

Since we began implementing the CES mental health response to COVID-19, we corroborated the importance of providing community-based mental health services. In many cases, CES services have been the only mental healthcare option for rural areas that could not access other services as these were unavailable. In addition, by providing mental healthcare through an all-female workforce of hired CMHWs, CES helps promote the creation of social and economic capital for women in these communities, ensures that the provided care is socio-culturally appropriate, and enables patients to benefit from the lived experiences of providers.

Globally, CHW programs are well established in many low-resource settings. Providing psychosocial support to communities through these professionals, who can also act as a liaison between community members and primary healthcare providers for more advanced mental health resources, may be a highly feasible alternative to respond to the reduced accessibility to mental health services and the increased burden of mental health issues due to the COVID-19 pandemic, as it can be more cost-effective than other alternatives [44,45]. Apart from our intervention, during the pandemic there have been other initiatives aimed at empowering communities to address mental health issues associated with the pandemic in LMICs, such as the BasicNeeds Pakistan initiative, which has trained over 3,600 community members as ‘Mental Health First Aiders’ [46], or the United Nations Refugee Agency (UNHCR) in Iraq, which provides psychosocial support to people living in refugee camps during the health emergency through CHWs, most of them refugees who provide PM+ psychotherapy [47].

Interventions should also be focused and tailored to groups at higher risk of developing mental health conditions due to COVID-19, such as women, adolescents, and frontline healthcare workers.

Studies from different areas of the world indicate that female gender is being significantly associated with worse mental health outcomes during the COVID-19 pandemic [48–51]. Women have a higher prevalence of risk factors for mental issues than men that have intensified during the virus outbreak, such as chronic environmental stress [52], preexisting prevalence of depressive and anxiety disorders [53], and intimate partner violence [54]. In addition, fear of perinatal complications due to COVID-19 infection or difficulties in accessing perinatal care have significantly increased the incidence of mental health issues among pregnant women worldwide during the pandemic [55,56]. This highlights the importance of implementing mental health interventions with a gender perspective. In our working context, many female patients have mentioned an increased workload; in addition to taking care of the household and children, women must now ensure that their children do their homework and that they care for family members with COVID-19. Additionally, the temporary suspension of some church activities has hampered women’s social networks, one of the few spaces where women socialize [14], which threatens to leave women living with domestic violence more isolated. Women are also the most affected by the economic stress associated with the pandemic [57], so it is essential to address gender-based social inequalities by improving women’s access to communication technologies and creating fair employment opportunities.

In relation to adolescents, different studies have identified more depressive and anxious symptoms compared to pre-pandemic estimates among this population around the globe [58]. In a poll conducted by UNICEF in Latin America and the Caribbean in mid-2021, 27% of adolescent and youth participants reported feeling symptoms of anxiety and 15% of depression in the seven days prior to the survey [59]. One of the main reasons behind these alarming figures is the fact that this population is under greater stress and suffers from a lack of activity due to the suspension of face-to-face school activities [60]. In the specific case of Chiapas, schools have opted to grade assignments that require searching the web, without considering that some adolescents have difficulty accessing mobile devices or covering the costs associated with internet access. This concern adds to a greater burden of responsibilities such as household chores or agricultural work. There is a global need to ameliorate the effects of the pandemic on the mental health of adolescents by improving the availability and accessibility of mental healthcare services for this population. This should be done through the collaboration of adolescents, their parents, health professionals, civil society actors and the government, and by reconciling epidemiological control measures with the specific needs of the adolescent population [60].

Worldwide, frontline healthcare professionals have suffered from a severe shortage of PPE and lack of comprehensive training in the care of COVID-19 patients, raising concerns about risk of infection, which has negatively impacted their mental health. In addition, the safety of their families and friends, the death of their colleagues, ethical concerns in clinical practice and excessive work hours have added to the detrimental effect of the pandemic on the mental wellbeing of these professionals [39,61]. Due to the impossibility of meeting these demands by overwhelmed public health sectors, the support of civil society organizations can be key in supporting the MoH in addressing them, as exemplified by our interventions and others from LMICs [62,63].

Finally, we also acknowledge that providing mental health care and health personnel capacity building during the pandemic in areas with poor access to digital devices and Information and Communications Technologies (ICTs) might be challenging and labor-intensive. To ensure participant safety, it has been necessary to limit several face-to-face mental health-related activities. However, in settings with good access to digital devices and ICTs, some of these activities can be adapted to be delivered remotely [64,65]. In our case, due to lack of internet access psychoeducation groups and patient involvement in behavioral activation activities had to be suspended. As for the health personnel training, poor internet connection resulted in having to facilitate several small group trainings in person.

From our work during the pandemic we conceived a broader idea of what kinds of interventions may be more appropriate to prevent and address the effects of the pandemic on people’s mental health in our context. However, the described intervention is limited to our context given the available resources, the barriers and the facilitators for implementing which might be diverse in different settings. For instance, the CES model relies on donations which are used to provide stipends for the staff and ensure the availability of supplies. This model also operates with highly motivated staff and ongoing training adapted to our particular context so that staff can acquire clinical competency. CES also has a successful collaboration with the MoH that allows operation within government facilities and collaboration with their staff. Hence, this paper is limited to describing and discussing our fieldwork experiences. We plan to assess the impact of our interventions in a future study that compares mental health outcomes in CES-supported areas with non-CES-supported areas during the pandemic period. This study will be the first to report on the effectiveness of mental health interventions in low-income rural communities with very limited access to digital devices and ICT during the COVID-19 pandemic and to report the experience of delivering mental health services within a COVID inpatient unit such as the RDC.

## Conclusions

There is a lack of mental health services for rural communities impacted by COVID-19 in Chiapas. Through a comprehensive strategy, CES addresses this gap and complements the available services created by the MoH, while caring for the wellbeing of the professionals who deliver the services. In marginalized communities, there is an urgent need to: 1) address the social determinants that increase their vulnerability to suffering from mental health conditions related to the pandemic, and 2) adapt mental health services to follow sanitary measures and be accessible to those living in rural and inaccessible areas with poor access to technology. In addition, psychosocial support is necessary to prevent and contain the mental health effects of the pandemic. Therefore, it is urgent that healthcare professionals have ongoing training in basic psychosocial support skills and for interventions to meet the needs of vulnerable populations – such as people with disabilities, older adults, children, and adolescents – that involve the voice of communities.

COVID-19 has made the need to prioritize mental health services more visible, and presents an opportunity to reimagine global mental health by building health systems using a task-sharing approach.

## Paper context

The mental health of marginalized populations has been most affected by the COVID-19 pandemic due to the limited human, economic, and social resources available. This article describes a public-civil partnership initiative to address the increased burden of mental illness in rural Mexico through capacity building and mental health care for health professionals and provision of psychosocial support for the general population. This is an opportunity to reimagine global mental health through comprehensive community-based initiatives.
